# A novel mycothiol-dependent thiol–disulfide reductase in *Corynebacterium glutamicum* involving oxidative stress resistance

**DOI:** 10.1007/s13205-021-02896-4

**Published:** 2021-07-14

**Authors:** Yang Liu, Xiaona Li, Jiaxin Luo, Tao Su, Meiru Si, Can Chen

**Affiliations:** 1 College of Life Sciences, Qufu Normal University, Qufu, 273165 Shandong China; 2 College of Life Science and Agronomy, Zhoukou Normal University, Zhoukou, 466001 Henan China

**Keywords:** Thiol–disulfide interchange protein (DsbA), *Corynebacterium glutamicum*, Oxidative stress, SigH

## Abstract

*ncgl2478* gene from *Corynebacterium glutamicum* encodes a thiol–disulfide oxidoreductase enzyme annotated as dithiol–disulfide isomerase DsbA. It preserves a Cys–Pro–Phe–Cys active-site motif, which is presumed to be an exclusive characteristic of the novel DsbA–mycoredoxin 1 (Mrx1) cluster. However, the real mode of action, the nature of the electron donor pathway and biological functions of NCgl2478 in *C. glutamicum* have remained enigmatic so far. Herein, we report that NCgl2478 plays an important role in stress resistance. Deletion of the *ncgl2478* gene increases the size of growth inhibition zones. The *ncgl2478* expression is induced in the stress-responsive extra-cytoplasmic function-sigma (ECF-σ) factor SigH-dependent manner by stress. It receives electrons preferentially from the mycothiol (MSH)/mycothione reductase (Mtr)/NADPH pathway. Further, NCgl2478 reduces *S*-mycothiolated mixed disulfides and intramolecular disulfides via a monothiol–disulfide and a dithiol–disulfide exchange mechanism, respectively. NCgl2478 lacks oxidase activity; kinetic properties of its demycothiolation are different from those of Mrx1. Site-directed mutagenesis confirms Cys24 is the resolving Cys residue, while Cys21 is the nucleophilic cysteine that is oxidized to a sulfenic acid and then forms an intramolecular disulfide bond with Cys24 or a mixed disulfide with MSH under oxidative stress. In conclusion, our study presents the first evidence that NCgl2478 protects against various stresses by acting as an MSH-dependent thiol–disulfide reductase, belonging to a novel DsbA–Mrx1 cluster.

## Introduction

*Corynebacterium glutamicum*, a well-known various l-amino acid producer in industrial applications and a model organism in systems biology, unavoidably generates or encounters a series of unfavorable circumstances in the fermenting process (Bröer et al. 1993). Various environmental insults, including oxidants, heavy metals, antibiotics, alkylating agents, and acids, induce accumulation of excessive reactive oxygen species (ROS) (Halliwell and Gutteridge 1984). These excessive ROS are highly reactive molecules that are not only capable of damaging cellular constituents such as DNA, RNA, lipids, and proteins, but also destroying intracellular redox homeostasis and provoking oxidative stress (Jiang et al. 2019; Storz et al. 1987). Thus, to survive within the diverse fermentation environment, *C. glutamicum* acquires a variety of mechanisms to protect its cellular constituents from ROS and maintain its redox equilibrium (Dalle-Donne et al. 2008). One of these mechanisms is the use of a low molecular weight (LMW) defense mechanism. In *C. glutamicum*, millimolar concentrations of MSH (mycothiol; chemically 1D-*myo*-inosityl-2-[*N*-acetyl-l-cysteinyl] amido-2-deoxy-α-d-glucopyranoside), a pseudodisaccharide containing a cysteine moiety as a reactive thiol, is the main LMW thiol involved in detoxification and maintaining redox homeostasis (Liu et al. 2013; Newton and Fahey 2008; Van Laer et al. 2012). The redox-active sulfydryl group of MSH protects cells from ROS by directly scavenging free radicals and by serving as a cofactor for antioxidant enzymes such as mycothiol peroxidase (MPx) and methionine sulfoxide reductase A (MsrA), in conjunction with mycoredoxin 1 (Mrx1) (Si et al. 2015a, b). Another mechanism is to use various antioxidative enzymes, including directly ROS-scavenging terminal enzymes such as catalase (KatG), superoxide dismutases (SOD), and peroxidase, oxidized proteins-repairing oxidoreductases, as well as regulatory proteins (Dalle-Donne et al. 2008).

Oxidized proteins-repairing oxidoreductases, such as thioredoxin (Trx), Mrx1, dithiol–disulfide isomerase (DsbA), DsbA-like Mrx1, and alkyl hydroperoxide reductase subunit D (AhpD), are believed to play a pivotal role in protecting against oxidative stress, maintaining intracellular thiol homeostasis, ensuring the proper folding of proteins and facilitating appropriate functioning of proteins in bacteria (Dalle-Donne et al. 2008; Su et al. 2019; Van Laer et al. 2012; Rosado et al. 2017). Mrx1 with the Cys–Pro–Tyr–Cys (C–P–Y–C) motif, an MSH-dependent disulfide oxidoreductase with a glutaredoxin-like sequence and function, reduces mixed disulfides between MSH and C_P_ of peroxidase with N-terminal cysteine of the active site via 1-Cys monothiol mechanism. Mrx1 is kept reduced with an NADPH-dependent flavoenzyme mycothione reductase (Mtr), MSH, and NADPH (Van Laer et al. 2012). DsbA with the Cys–Pro–His–Cys (C–P–H–C) motif usually acts as an oxidase that catalyzes the oxidative refolding of RNase I (Greiner-Stoeffele et al. 1996). Recently, Rosado et al. (2017) found *C. glutamicum* NCgl2339 with the Cys–Pro–Phe–Cys (C–P–F–C) motif and *Mycobacterium tuberculosis* Rv2466c with the Cys–Pro–Trp–Cys (C–P–W–C) motif had the activity of demycothiolating and reducing a mycothiol arsenate adduct. NCgl2339 and Rv2466c have no oxidase properties like classic DsbAs. Moreover, their kinetic properties were different from those of classic Mrx1. Therefore, Rosado et al. believed NCgl2339 and Rv2466c were novel oxidoreductases, belonging to DsbA-like Mrx1. However, some aspects of this new type of oxidoreductase are poorly understood so far, such as their mode of action, actual enzymatic functioning, and biological function. Thus, an in-depth analysis of the physiological and biochemical characteristics and catalytic mechanisms is vital. Bioinformatics analysis reveals that *C. glutamicum* NCgl2478 preserves the C–P–F–C active-site sequence motif, similar to that of NCgl2339. The phenomenon indicated NCgl2478 may be a potentially new redox enzyme and protect *C. glutamicum* from oxidative stress. In this study, we selected ORF NCgl2478 and sought to explore the physiological and biochemical functions of NCgl2478 in *C. glutamicum*, paving the way for correctly classifying similar enzymes from other organisms.

## Material and methods

### Bacterial strains and growth conditions

The bacterial strains and plasmids used in this study were listed in Table 1. *C. glutamicum* RES167 strains used in this study were derived from the sequenced strain ATCC 13032*. Escherichia coli* JM109 (Stratagene, United States) and *C. glutamicum* RES167 strains were grown on either Luria–Bertani (LB) broth or LB agar plates at 37 and 30 °C under vigorous agitation (220 rpm) as previously reported, respectively (Shen et al. 2005). To produce and maintain mutant of a gene in *C. glutamicum*, brain–heart broth medium containing 0.5 M sorbitol (BHIS) was used (Shen et al. 2005). *ncgl2478* gene in-frame deletion was generated using the method described (Shen et al. 2005). For complementation, the pXMJ19-*ncgl2478* derivatives were transformed into Δ*ncgl2478* mutant by electroporation. The transformant was selected on nalidixic acid and chloramphenicol-containing LB plates and its expression in *C. glutamicum* was induced by adding 0.5 mM isopropyl β-d-1-thiogalactopyranoside (IPTG) (Sigma-Aldrich) (Shen et al. 2005). Antibiotics were added at the following concentrations: kanamycin, 50 µg ml^−1^ for *E. coli* and 25 µg ml^−1^ for *C. glutamicum*; nalidixic acid, 40 µg ml^−1^ for *C. glutamicum*; chloramphenicol, 20 µg ml^−1^ for *E. coli* and 10 µg ml^−1^ for *C. glutamicum*.

**Table 1:** Bacterial strains and plasmids used in this study

Strains or plasmids	Relevant genotype description	Source or references
Strains		
* Corynebacterium glutamicum*		
RES167	Restriction-deficient mutant of ATCC13032, Δ(*cglIM–cglIR–cglIIR*)	Tauch et al. (2002)
Δ*ncgl2478*	*ncgl2478* deleted in RES167	This study
Δ*sigH*		Si et al. (2014a)
* E. coli*		
BL21(DE3)	*E. coli* expression host, *hsdS gal* (*λc*I*ts*857 *ind-l Sam7 nin-*5 *lac UV5-*T7 gene 1)	Novagen
JM109	*recA1 supE44 endA1 hsdR17 gyrA96 relA1 thi* Δ(*lac-proAB*)F′(*traD36 proABlacI*^q^ *lacΔZM15*)	Stratagene (catalog no. 200235)
Plasmids		
pK18*mobsacB*	Suicide plasmid carrying *sacB* for selecting double crossover in *C. glutamicum*, Km^r^	Schäfer et al. (1994)
pK18*mobsacB-*Δ*ncgl2478*	Construct used for in-frame deletion of *ncgl2478*	This study
pK18*mobsacB-P*_*ncgl2478*_*::lacZY*	*P*_*ncgl2478*_*::lacZY* fusion in pK18*mobsacB*	This study
pXMJ19	Shuttle vector (*P*_*tac*_* lacI*^*q*^* pBL1 oriV*_*C. glutamicum*_ pK18 *oriV*_*E. coli*_)	Jakoby et al. (1999)
pXMJ19-*ncgl2478*	*ncgl2478* cloned into pXMJ19 for complementation	This study
pET28a	Expression vector with N-terminal hexahistidine affinity tag	Novagen
pET28a*-ncgl2478*	*ncgl2478* in pET28a	This study
pET28a-*ncgl2478:C21S*	*ncgl2478:C21S* in pET28a	This study
pET28a-*ncgl2478:C24S*	*ncgl2478:C24S* in pET28a	This study
pET28a*-mrx1*		Si et al. (2014b)
pET28a*-mrx1:C15S*		Si et al. (2014b)
pET28a*-mtr*		Si et al. (2014b)
pET28a*-trx*		Si et al. (2014b)
pET28a*-trxR*		Si et al. (2014b)
pET28a*-sigH*		Si et al. (2014a)

### Plasmid construction

Primers used in this study were listed in Table 2. The *ncgl2478* gene region of *C. glutamicum* was amplified with primer pair ONCgl2478-F and ONCgl2478-R from genomic DNA of *C. glutamicum RES167* by PCR, and then the resulting fragments cut with *Bam*HI and *Hin*dIII enzymes were cloned into appropriately digested pET28a to give plasmids pET28a-*ncgl2478*. The suicide plasmid pK18*mobsacB*-Δ*ncgl2478* was obtained by two-step recombination as described previously (Su et al. 2018). To prepare pXMJ19-*ncgl2478*, primer pair CNCgl2478-F/CNCgl2478-R was used to amplify the *ncgl2478* gene DNA fragments from *C. glutamicum* genomic DNA. The resulting DNA fragments were cut and then cloned into similar digested pXMJ19. To generate pET28a-*ncgl2478:C21S* or pET28a-*ncgl2478:C24S*, site-directed mutagenesis was performed by two rounds of PCR as described (Si et al. 2015a, 2018a). To obtain the *lacZY* fusion reporter vector pK18*mobsacB-P*_*ncgl2478*_*::lacZY*, overlap PCR was performed to fuse the *ncgl2478* promoter to the *lacZY* reporter gene (Su et al. 2018). For obtaining pK18*mobsacB-P*_*ncgl2478M*_*::lacZY*, 258-bp *ncgl2478* promoter DNA containing the mutagenized sequence of the predicted stress-responsive extra-cytoplasmic function-sigma (ECF-σ) factor SigH binding site (*P*_*ncgl2478M*_) was first directly synthesized by Shanghai Biotechnology Co., Ltd. Start and stop sites of *P*_*ncgl2478M*_ were the same as those of *P*_*ncgl2478*_ in *P*_*ncgl2478*_*::lacZY*. Then, the resulting 258-bp *P*_*ncgl2478M*_ was fused to a *lacZY* reporter gene*.* Finally, *P*_*ncgl2478M*_*::lacZ* was inserted into similarly digested pK18*mobsacB.* The fidelity of all constructs was confirmed by DNA sequencing (Sangon Biotech, Shanghai, China).

**Table 2:** Primers used in this study

Primiers	5′–3′ sequence	
Cncgl2478-F1	CCCAAGCTTATGTCTATTGAATTCTCCGCAC (*Hin*dIII)	For cloning *ncgl2478* wild type and mutants into pXMJ19
Cncgl2478-R1	CGCGGATCCTTAGTTGCAGGTGCCGTCGACG (*Bam*HI)	
Oncgl2478-F	CGCGGATCCATGTCTATTGAATTCTCCGCAC (*Bam*HI)	For cloning *ncgl2478* wild type and mutants into pET28a
Oncgl2478-R	CCCAAGCTTTTAGTTGCAGGTGCCGTCGACG (*Hin*dIII)	
Dncgl2478-F1	GGAAGATCTCTATGACATGATTACGAATTCGTGATGATTTCCGGTTCGTCGAC (*Bgl*II)	To generate pK18*mobsacB-*Δ*ncgl2478*
Dncgl2478-R1	CCGATGTAGCAGAAGGGGCACATG	
Dncgl2478-F2	CATGTGCCCCTTCTGCTACATCGGCAGCCCATTTGAGGTCATTGAC	
Dncgl2478-R2	ACGCGTCGACTGCTTGGTATTCAAAAAGATCCAC (*Sal*I)	
Oncgl2478-C21S-F	GGAGCGACATCATG*A*GCCCCTTCTGCTAC	To generate *ncgl2478:C21S* DNA fragment
Oncgl2478-C21S-R	GTAGCAGAAGGGGC*T*CATGATGTCGCTCC	
Oncgl2478-C24S-F	GACATCATGTGCCCCTTC*A*GCTACATCGGCAAAAAG	To generate *ncgl2478:C24S* DNA fragment
Oncgl2478-C24S-R	CTTTTTGCCGATGTAGC*T*GAAGGGGCACATGATGTC	
*Pncgl2478*-F	TCCCCCGGGATCTTAAGTACCCCTGTTTTGGAG (*Sma*I)	To generate pK18*mobsacB-P*_*ncgl2478*_*::lacZY* and the 258-bp *ncgl2478* promoter
*P*_*ncgl2478*_-R	ACTAGTTAGCCCATGCACCACGATAACGCG (*Spe*I)	
lacZY-F1	CGCGTTATCGTGGTGCATGGGCTAACTAGT ATGACCATGATTACGGATTC(*Spe*I)	
lacZY-R	AAAACTGCAGTTAAGCGACTTCATTCACCTG(*Pst*I)	
Qncgl2478-F	CGCCAAATGAACGGCCAAGTCC	RT-PCR
Qncgl2478-R	GGCTTTCGCGAAGTGGGTAAGG	
Encgl2478-F	GTACCCCTGTTTTGGAGAATGC	To produce the 211-bp EMSA promoter DNA
Encgl2478-R	CGTCTCCTTGCAATGTGAACCC	
Control-F	TGACGAAACCATCGCGGCCAACAC	To produce the 211-bp EMSA control DNA
Control-R	CACGTCGGATTCGAGAGCTTCGCG	
16 S rRNA-F	ACCCTTGTCTTATGTTGCCAG	RT-PCR
16 S rRNA-R	TGTACCGACCATTGTAGCATG	
F1-F	GGCCGTTCATTTGGCGGGCCTGCTC	Co-transcription
F1-R	CCAAAATAACCTGGGGTTTCCTCGC	

Underlined sites indicated restriction enzyme cutting sites added for cloning. Letters in italic denoted the mutation sites in overlap PCR for site-directed mutation

### Overexpression and purification of recombinant protein

pET28a derivatives were transformed into *E. coli* BL21(DE3) cells and recombinant proteins were purified as described previously (Si et al. 2018b). Eluted recombinant His_6_-NCgl2478 proteins were concentrated and loaded onto a Superdex-75 10/300 gel filtration column (GE Healthcare, Piscataway, NJ) with a running condition of 10 mM Tris (pH 7.4), 100 mM NaCl, and 5 mM β-mercaptoethanol. For conducting subsequent enzyme activity experiments, the His_6_ tag in protein was cut in the presence of 10 units of Enterokinase-Max (Invitrogen, Karlsruhe, Germany) at 4 °C overnight. To remove the cleaved tag and uncleaved protein, Ni–NTA agarose was used. All enzymes were purchased from Sigma-Aldrich (St. Louis, MO). Resulting His_6_-tag-free protein was dialyzed against PBS at 4 °C and concentrated for further experiments [> 95% purity as estimated by sodium dodecyl sulfate–polyacrylamide gel electrophoresis (SDS-PAGE)].

### Agar-based disk diffusion assay

Disk diffusion assays were performed for alkylating agents and oxidative agents according to Rawat et al. (2002). Briefly, bacterial strains were grown to the mid-log phase and 100 μl of culture containing about 10^7^ CFU was spread onto 20 ml LB agar plates. Paper disks soaked with 10 μl of a stock solution of reagents were placed on top of the agar. Stock solutions were 50 mM hydrogen peroxide (H_2_O_2_), 5.5 mM cumene hydroperoxide (CHP), 0.2 mM sodium hypochlorite (NaClO), 5 mM diamide, 50 mM 2, 4-dinitrochlorobenzene (DNCB), and 0.6 mM iodoacetamide (IAM). The disks were allowed to dry and the plates were incubated for 2–3 days at 30 °C. The diameter of the inhibition zones was measured. Experiments were performed in triplicate.

### Electrophoretic mobility shift assay (EMSA)

EMSA was performed according to the method described previously (Su et al. 2019).

### Preparation of oxidized NCgl2478-S_2_ in vitro

Oxidized NCgl2478-S_2_ was prepared according to previously described (Van Laer et al. 2012; Pedre et al. 2015) protocol. First, NCgl2478 or Mrx1 from *C. glutamicum* was reduced by incubation with 50 mM DTT for 30 min at room temperature. Second, excess DTT was removed by ultrafiltration. Third, pre-reduced NCgl2478 or Mrx1 was oxidized with a fivefold molar excess of diamide and incubated for 30 min at room temperature. Finally, oxidized NCgl2478-S_2_ or Mrx1-S_2_ was purified on a Superdex 75 10/300 GL column (GE Healthcare) equilibrated with 20 mM Tris, 150 mM NaCl, pH 7.6 for further experiments. Pure NCgl2478-S_2_ and Mrx1-S_2_ were confirmed by Matrix-Assisted Laser Desorption/Ionization Time of Flight Mass Spectrometry (MALDI-TOF MS).

### Steady-state kinetics of oxidized NCgl2478-S_2_ by Trx/TrxR/NADPH and MSH/Mtr/NADPH pathway

Oxidized NCgl2478-S_2_-dependent oxidation of NADPH in the Trx/TrxR/NADPH or MSH/Mtr/NADPH pathway was continuously monitored at 340 nm (*ε*_340_ = of 6220 M^−1^·cm^−1^) in reaction mixture. In the Trx/TrxR/NADPH pathway, the reaction mixture contained 50 mM Tris–HCl buffer (pH 7.5), 1 mM EDTA, 5 μM *C. glutamicum* TrxR, 300 μM NADPH, 5 μM *C. glutamicum* Trx, and varying concentrations of oxidized NCgl2478-S_2_. In the MSH/Mtr/NADPH pathway, reaction mixture contained 50 mM Tris–HCl buffer (pH 7.5), 1 mM EDTA, 5 μM *C. glutamicum* Mtr, 300 μM NADPH, 500 μM MSH, and varying concentrations of oxidized NCgl2478-S_2_. All reactions were carried out at 37 °C and started with the addition of oxidized NCgl2478-S_2_ in a reaction mixture previously incubated for 3 min at 37 °C. Control measurements were performed in the absence of NCgl2478-S_2_. Reactions were performed in duplicate. Mrx1-S_2_ was used as a positive control. The *k*_cat_ and *K*_m_ values were obtained from a non-linear fit with the Michaelis–Menten equation using the program GraphPad Prism 5.

### Quantitative analysis of sulfhydryl groups

Free sulfhydryl groups in wild-type NCgl2478 (NCgl2478 WT) and its variants were measured using 5, 5′–dithio–bis (2–nitrobenzoic acid) (DTNB) (Ellman 1959). The amounts of reactive sulfhydryl groups were measured using the molar absorption coefficient of TNB at 412 nm (*ε*_412_) of 13,600 M^−1^·cm^−1^ (Gething and Davidson 1972).

### NBD–Cl analysis of the sulfenic acid state

To study the formation of cysteine sulfenic acid (Cys–SOH) as a reaction intermediate, NCgl2478:C21S and NCgl2478:C24S labeled with 4-chloro-7-nitrobenzofurazan (NBD–Cl) were assayed as described previously (Selles et al. 2012).

### Enzymatic activity assay

Insulin disulfide reduction was performed based on the method described by Rosado et al. (2017). The precipitation starting point was defined as an increase of 0.02 absorbance units at *A*_600_ after a stable baseline recording and the rate of precipitation was calculated using a linear regression composed of *A*_600_ ranging from 2000 to 2500 s (Holmgren 1979).

Hydroxyethyl disulfide (HED) was utilized to prepare and measure the mixed disulfide between MSH and 2-hydroxyethyl disulfide (HED) (HED-SSM) reduction activity (Si et al. 2019). HED-SSM was formed by incubating 1000 mM HED with 50 mM MSH at 30 °C for 3 min. Briefly, the kinetic parameters were determined in the presence of varying concentrations of HED-SSM (0–20 mM). The enzyme reactions were measured in 50 mM potassium phosphate buffer (pH 7.6), 250 μM NADPH, 5 μM Mtr, 500 μM MSH, and 1 μM NCgl2478 (WT, C21S, or C24S). The assay was performed at 25 °C. Absorption was monitored at 340 nm. The activity was determined after subtracting the spontaneous reduction rate observed in the absence of NCgl2478 and the number of micromoles of NADPH oxidized per second per micromole of enzyme (i.e. the turnover number, s^−1^) was calculated using the molar absorption coefficient of NADPH at 340 nm (*ε*_340_) of 6220 M^−1^·cm^−1^. Three independent experiments were performed at each substrate concentration. The *k*_cat_ and *K*_m_ values of NCgl2478 for HED-SSM substrates were obtained from a non-linear fit with the Michaelis–Menten equation using the program GraphPad Prism 5. Mrx1 was used as a control.

### p*Ka* determination

The extinction coefficient of thiol groups (R-SH) at 240 nm was the main readout utilized to measure p*Ka* values of cysteine residues due to the lack of absorption of its un-ionized counterpart (R-S^−^) in the same wavelength (Roos et al. 2013). To cover a broad pH range, a reaction mixture containing a poly-buffer solution composed of 10 mM sodium acetate, 10 mM sodium phosphate, 10 mM sodium borate, and 10 mM sodium citrate, pH 9.4, was used. For the oxidation of cysteine mutants, a tenfold excess of H_2_O_2_ was used. Excess H_2_O_2_ was removed by ultrafiltration. A final reaction mixture of 20 μM NCgl2478 (reduced or oxidized) was titrated with 100 mM HCl. The p*Ka* of NCgl2478:C22S and NCgl2478:C24S variants were determined in the same conditions as described for NCgl2478 WT. The measurements were performed in a Carry UV spectrophotometer (Agilent Technologies) precooled to 10 °C. The sigmoidal pH-dependent saturation curve was fitted to the Henderson-Hasselbalch equation (Roos et al. 2007), where *A*_exp_ was the experimental value*A*_240_/*A*_280_, *A*_SH_ was the *A*_240_/*A*_280_ value for the protonated form, and *A*_S_^−^ is the *A*_240_/*A*_280_ for the deprotonated form. The data were fitted to the following equation using GraphPad Prism version 5 (San Diego California USA).

$$A_{{\exp }} = A_{{{\text{SH}}}} + \frac{{\left( {A_{{{\text{S}}^{ - } }} - A_{{{\text{SH}}}} } \right)}}{{1 + 10^{{\left( {p{\text{K}}_{{\text{a}}} - {\text{pH}}} \right)}} }}$$

### RNase I activity assay

Oxidase activity was measured as described previously (Roos et al. 2007).

#### Construction of chromosomal fusion reporter strains and β-Galactosidase assay

The *lacZY* fusion reporter plasmid pK18*mobsacB-P*_*ncgl2478*_*::lacZY* was transformed into the corresponding *C. glutamicum* RES 167 strain by electroporation and the chromosomal fusion reporter strains were selected on LB agar plates supplemented with kanamycin (Si et al. 2019). The resulting strains were grown in LB medium to an optical density at 600 nm of 0.6–0.7 and then treated with different reagents of various concentrations at 30 °C for 30 min. β-Galactosidase activity was assayed with *O*-nitrophenyl-β-d-galactopyranoside (ONPG) as the substrate (Miller 1992). The β-Galactosidase data presented were the averages of three independent biological experiments and error bars indicated the SDs from three independent experiments.

### Quantitative real-time polymerase chain reaction (qRT-PCR) analysis

Isolation of the total RNA and transcript levels analysis was performed as described previously (Si et al. 2019). To obtain standardized results, the relative abundance of the 16S rRNA gene was used as the internal standard.

### Statistical analysis

The results shown represented the average of three independent experiments; error bars indicated the standard deviation (SD) from three independent experiments. Statistical analyses of survival rate, transcription level, and protein level were determined with paired two-tailed Student’s *t* test. GraphPad Prism Software was used to carry out statistical analyses (GraphPad Software).

## Results and discussion

### *C. glutamicum* NCgl2478 null mutant was sensitive to oxidative stress

**Fig. 1:**
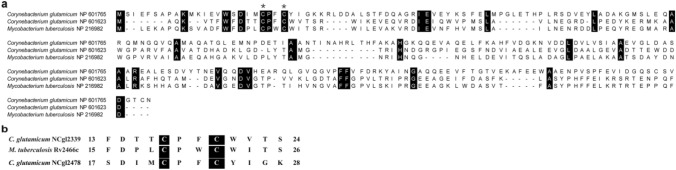
Multiple sequence alignment of NCgl2478 with Rv2466c from *M. tuberculosis* and NCgl2339 from *C. glutamicum*. **a** Active site Cys was pointed out by a black star. Reference sequences were retrieved from the NCBI Database, including *C. glutamicum* ATCC NCgl2478 (NP_601765), *C. glutamicum* ATCC NCgl2339 (NP_601623); *Mycobacterium tuberculosis* Rv2466c (NP_216982). **b** Sequence alignment between active-site cysteines in NCgl2478 and Rv2466c from *M. tuberculosis* or NCgl2339 from *C. glutamicum*. Magenta highlights the dicysteine motif

*C. glutamicum* NCgl2478 containing the Cys–Pro–Phe–Cys (C–P–F–C) motif is annotated as a dithiol-disulfide isomerase DsbA. Recently, a demonstration indicated that Rv2466c from *M. tuberculosis* and NCgl2339 from *C. glutamicum*, having high sequence similarity to DsbA, belong to a novel DsbA–Mrx1 cluster (Rosado et al. 2017). The novel DsbA–Mrx1 cluster has the special Cys–Pro–Trp/Phe–Cys (C–P–W/F–C) active-site sequence motif and exhibits different enzymatic features and substrate preferences from Mrx1 cluster with the catalytic motif Cys–Pro–Tyr–Cys (C–P–Y–C) or classical DsbA cluster with the catalytic motif Cys–Pro–His–Cys (C–P–H–C) (Rosado et al. 2017). Although amino acid sequence comparison revealed NCgl2478 is only 29.8% identical to Rv2466c and 26.2% identical to NCgl2339 (Fig. 1a), we presumed it might be also a member of the DsbA-Mrx1 cluster involved in stress response as it shared the C–P–F–C signature motif presumed to be an exclusive characteristic of the DsbA–Mrx1 cluster (Fig. 1b). *M. tuberculosis* Rv2466c is validated to promote mycobacterial resistance to oxidative stress (Rosado et al. 2017). Thus, to assess the role of NCgl2478 in protecting cells against oxidative stress, we constructed an *ncgl2478* null mutant in *C. glutamicum* and tested the sensitive phenotype of *ncgl2478* mutant to various oxidizing and H_2_O_2_-inducing agents [hydrogen peroxide (H_2_O_2_), cumene hydroperoxide (CHP), sodium hypochlorite (NaClO), diamide, 2, 4-dinitrochlorobenzene (DNCB), and iodoacetamide (IAM)] by an agar-based disc diffusion assay. As shown in Table 3, Δ*ncgl2478* strain (the mutant lacking *ncgl2478* with the empty plasmid pXMJ19) showed decreased resistance to all chemical reagents tested challenge compared to the WT strain (the *C. glutamicum* RES167 strain with the empty plasmid pXMJ19), giving a significantly larger zone of inhibition than WT strain. To confirm that the sensitivity to reagents may occur when lacking *ncgl2478*, complementary strain Δ*ncgl2478*^+^ was constructed by the introduction of plasmid pXMJ19 *in trans* containing the wild-type *C. glutamicum ncgl2478* gene into *ncgl2478* null mutant. As shown in Table 3, sensitive phenotypes were almost fully restored in Δ*ncgl2478*^+^. Although deletion of *ncgl2478* did not affect *C. glutamicum* growth under normal condition, NCgl2478 was important for survival under various oxidative stress conditions.

**Table 3:** Sensitivity of *C. glutamicum* strains to oxidizing agents tested by disk diffusion assay

Agents	Size of growth inhibition zone (cm) of various strains^a^		
	WT	Δ*ncgl2478*	Δ*ncgl2478*^+^
H_2_O_2_	1.5 ± 0.3	2.7 ± 0.5**	1.6 ± 0.2
CHP	1.8 ± 0.3	2.3 ± 0.4*	1.7 ± 0.4
NaClO	2.7 ± 0.2	3.2 ± 0.3*	2.8 ± 0.4
Diamide	1.4 ± 0.3	1.8 ± 0.2*	1.5 ± 0.2
DNCB	1.6 ± 0.2	2.1 ± 0.3*	1.7 ± 0.4
IAM	1.7 ± 0.4	2.2 ± 0.3*	1.7 ± 0.2

*H*_*2*_*O*_*2*_ hydrogen peroxide, *CHP* cumene hydroperoxide, *NaClO* sodium hypochlorite, *DNCB* 2,4-dinitrochlorobenzene, *IAM* iodoacetamide

*P ≤ 0.05 or **P ≤ 0.01 versus WT for the Δ*ncgl2478* mutant

^a^The values were mean ± SD for three independent determinations

### Formation of an intramolecular disulfide bond Cys21–Cys24 under oxidative stress

**Fig. 2:**
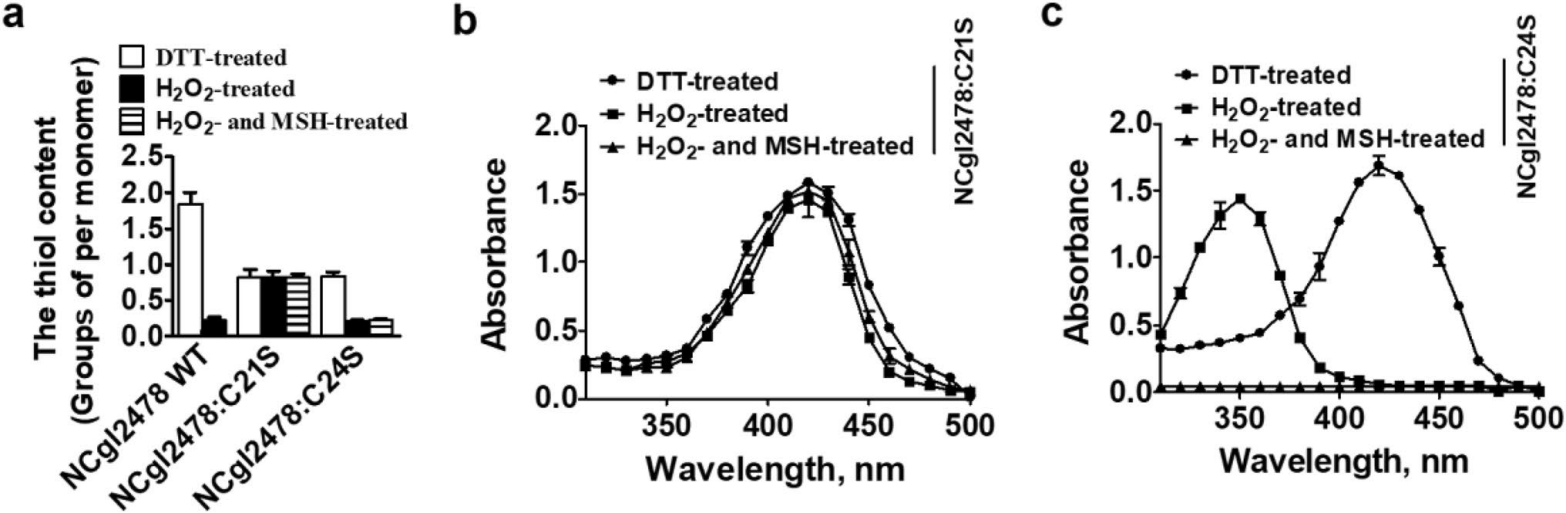
The thiol content of DTT- or H_2_O_2_-treated NCgl2478. **a** Free sulfhydryl groups in NCgl2478 WT and its variants were determined using 5, 5′-dithio-bis (2-nitrobenzoicacid) (DTNB). **b**, **c** Spectrophotometric analysis of NBD-labelled NCgl2478:C21S and NCgl2478:C24S. Reduced proteins treated with and without H_2_O_2_ or H_2_O_2_ and MSH were modified with NBD–Cl for 30 min. The resulting proteins were analyzed spectrophotometrically at 200–600 nm

The Cys in the Cys^19^–Pro–Trp–Cys^22^ (C^19^–P–W–C^22^) active-site motif of *M. tuberculosis* Rv2466c could form Cys19–Cys22 disulfide and Cys19–MSH mixed disulfide under oxidative stress (Rosado et al. 2017). Moreover, Cys19 and Cys22 of *M. tuberculosis* Rv2466c were the nucleophilic and resolving cysteines, respectively. Amino acid sequence comparison showed that Cys21 of NCgl2478 might be the nucleophilic cysteine residue (C_p_) that had been reported to be involved in catalysis via the transient formation of a sulfenic acid (Cys–SOH) (Baker and Poole 2003) (Fig. 1a). However, Cys24 might be the resolving Cys residues (C_R_). During catalysis, the labile peroxidatic Cys–SOH is easily attacked by C_R_ to form the redox-active disulfide. To trap Cys–SOH, the C-terminal cysteine of the active-site disulfide pair must be removed. Therefore, to investigate whether Cys21 and Cys24 have the above speculative function and test if these two cysteine residues in NCgl2478 could undergo disulfide after oxidation, we mutated the first and the second cysteine of the CXXC motif to serine, and these two mutated proteins NCgl2478:C21S and NCgl2478:C24S were purified by the Ni–NTA His·Bind Resin. NCgl2478 WT, NCgl2478:C21S and NCgl2478:C24S with and without previous exposure to H_2_O_2_ were used to carry out the free thiol content analysis. As shown in Fig. 2a, the DTT-treated NCgl2478 WT contained 1.85 ± 0.47 thiol groups per monomer, but the thiol content decreased to 0.22 ± 0.06 when NCgl2478 WT was treated with H_2_O_2_. The difference of 1.63 thiol groups between the two preparations was linked to the full oxidation of NCgl2478 WT after H_2_O_2_ treatment. These data indicated that NCgl2478 WT was fully oxidized by H_2_O_2_ to form a disulfide bond between Cys21 and Cys24.

NBD-Cl can exclusively react with free thiol groups in protein and protein sulfenic acids (P‐SOH), but not with protein sulfinic (P‐SO_2_H) and sulfonic (P‐SO_3_H) acid. The covalent attachment of NBD-Cl generated an absorption peak at ∼ 420 nm upon reaction with thiol groups, whereas it peaked at ∼ 347 nm upon reaction with sulfenic acids (Baker and Poole 2003). Following reaction with NBD–Cl, the absorption spectra of the NCgl2478:C21S variants remained unchanged before and after exposure to H_2_O_2_ or H_2_O_2_ and MSH, exhibiting only the 420 nm peak (Fig. 2b). DTNB assay for free thiol contents also showed one thiol per monomer before and after H_2_O_2_ or H_2_O_2_ and MSH treatment, implying no SOH is formed on Cys24, and Cys24 was still in thiol form under exposure to H_2_O_2_ or H_2_O_2_ and MSH (Fig. 2a). However, NCgl2478:C24S under H_2_O_2_ treatment lost one thiol group, compared to the thiol content of DTT-treated state, indicating that Cys21 did not exist as a thiol in H_2_O_2_-treated NCgl2478:C24S variant (Fig. 2a). Consistently, H_2_O_2_-treated and NBD-labeled NCgl2478:C24S had an absorbance maximum (*λ*_max_) of 347 nm, representing the NBD-modified product Cys–S(O)–NBD (Ellis and Poole 1997), which clearly signified the detection and trapping of SOH at Cys21, the only Cys in the NCgl2478:C24S variant (Fig. 2c). However, non-H_2_O_2_-treated NCgl2478:C24S modified with NBD–Cl produced a new covalently attached spectral species with a *λ*_max_ of 420 nm, consistent with previously characterized thiol adducts with NBD-Cl (Cys–S–NBD). Interestingly, no NBD–Cl labeling in H_2_O_2_-treated NCgl2478:C24S occurred in the presence of MSH, indicating MSH reacted with NCgl2478:C24S–SOH to form NCgl2478:C24S–SSM (the mixed disulfide between MSH and NCgl2478:C24S). This result showed that Cys21 was sensitive to oxidant, forming a sulfenic acid (Cys21–SOH). In the presence of MSH, MSH directly interacted with Cys21–SOH to form Cys21–MSH mixed disulfide. Further, Cys24 resolved the Cys21–MSH mixed disulfide or Cys21–SOH, leading to the formation of a Cys21–Cys24 disulfide. This result agrees with what Rosado et al. reported for *M. tuberculosis* Rv2466c (Rosado et al. 2017).

### Oxidized NCgl2478 preferred the MSH/Mtr/NADPH pathway as an electron source

**Fig. 3:**
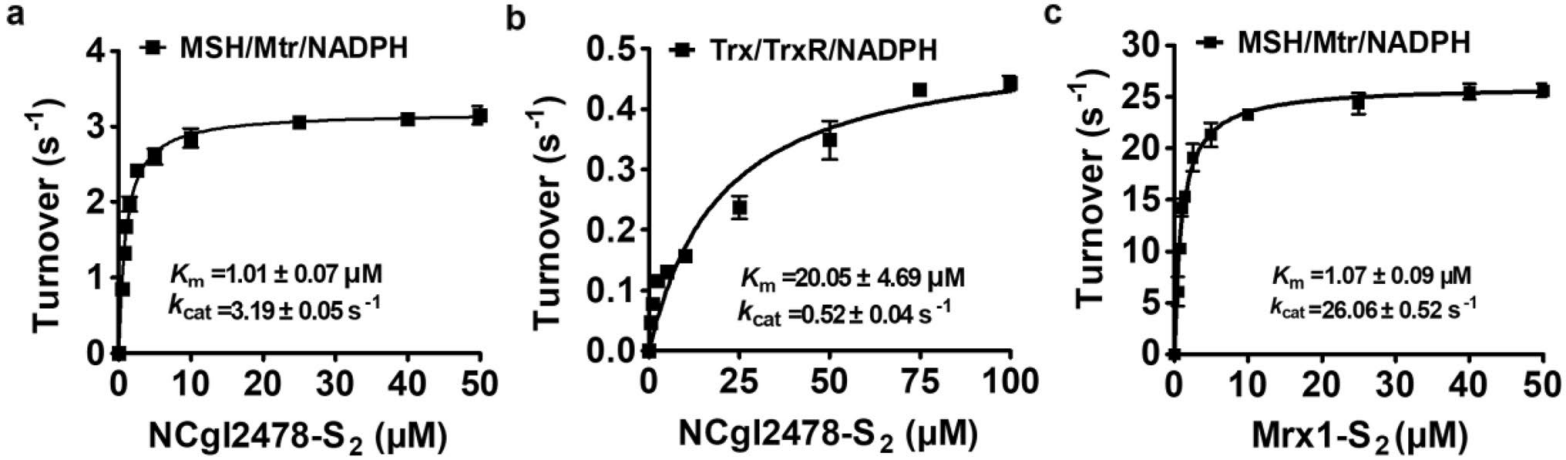
Oxidized NCgl2478–S_2_ was reduced preferably by the MSH/Mtr/NADPH pathway. The reduction of the oxidized NCgl2478–S_2_ (**a**) and Mrx1–S_2_ (**c**) by the MSH/Mtr/NADPH, or the reduction of the oxidized NCgl2478–S_2_ (**b**) by the Trx/TrxR/NADPH pathway was evaluated via Michaelis–Menten steady-state kinetics using the program GraphPad Prism 5. The data were represented as mean ± SD of three independent experiments. Different concentrations of oxidized NCgl2478–S_2_ or Mrx1–S_2_ were mixed with a pre-incubated mixture of the MSH, Mtr, and NADPH, or Trx, TrxR, and NADPH

To identify possible electron donor pathways coupled to NCgl2478 reduction, two of the most important electron transfer pathways in *C. glutamicum* responsible for keeping the redox potential in balance were reproduced in vitro, that of the MSH/Mtr/NADPH and Trx/TrxR/NADPH. To do so, NCgl2478 was first oxidized with a fivefold molar excess of diamide to obtain NCgl2478–S_2_ (NCgl2478ox). NCgl2478–S_2_ with a single disulfide bond between its active site cysteines was added as a substrate for the two electron transfer pathways mentioned above to measure steady-state kinetics. As shown in Fig. 3a, b, the *K*_m_ value, *k*_cat_ value, and catalytic coefficient of NCgl2478-S_2_ for the MSH/Mtr/NADPH or the Trx/TrxR/NADPH electron donor pathway were calculated to be 1.01 ± 0.07 μM, 3.19 ± 0.05 s^−1^, and 3.15 ± 0.07 × 10^6^ M^−1^ s^−1^, or 20.05 ± 4.69 μM, 0.52 ± 0.04 s^−1^, and 2.57 ± 0.38 × 10^4^ M^−1^ s^−1^, respectively. It is worth noting that reduction of the oxidized form of Mrx1 (Mrx1ox) through the MSH/Mtr/NADPH pathway resulted in a catalytic coefficient of 2.43 × 10^7^ M^−1^ s^−1^ (Fig. 3c), and Mrx1 has been shown previously not to use the Trx/TrxR/NADPH electron donor pathway (Van Laer et al. 2012). Here, we have clearly shown that although NCgl2478ox could be reduced by both electron pathways, the enzyme was reduced about 120-times faster with the MSH/Mtr/NADPH pathway compared with the Trx/TrxR/NADPH pathway, indicating NCgl2478ox prefers the MSH/Mtr/MSH pathway. Mrx1ox catalyzed an approximately eightfold-faster reaction coupled to the MSH/Mtr/NADPH pathway compared with NCgl2478ox, indicating a different specificity of electron donor pathway for both enzymes (Fig. 3a, c).

### The p*Ka* of the cysteine residues

**Fig. 4:**
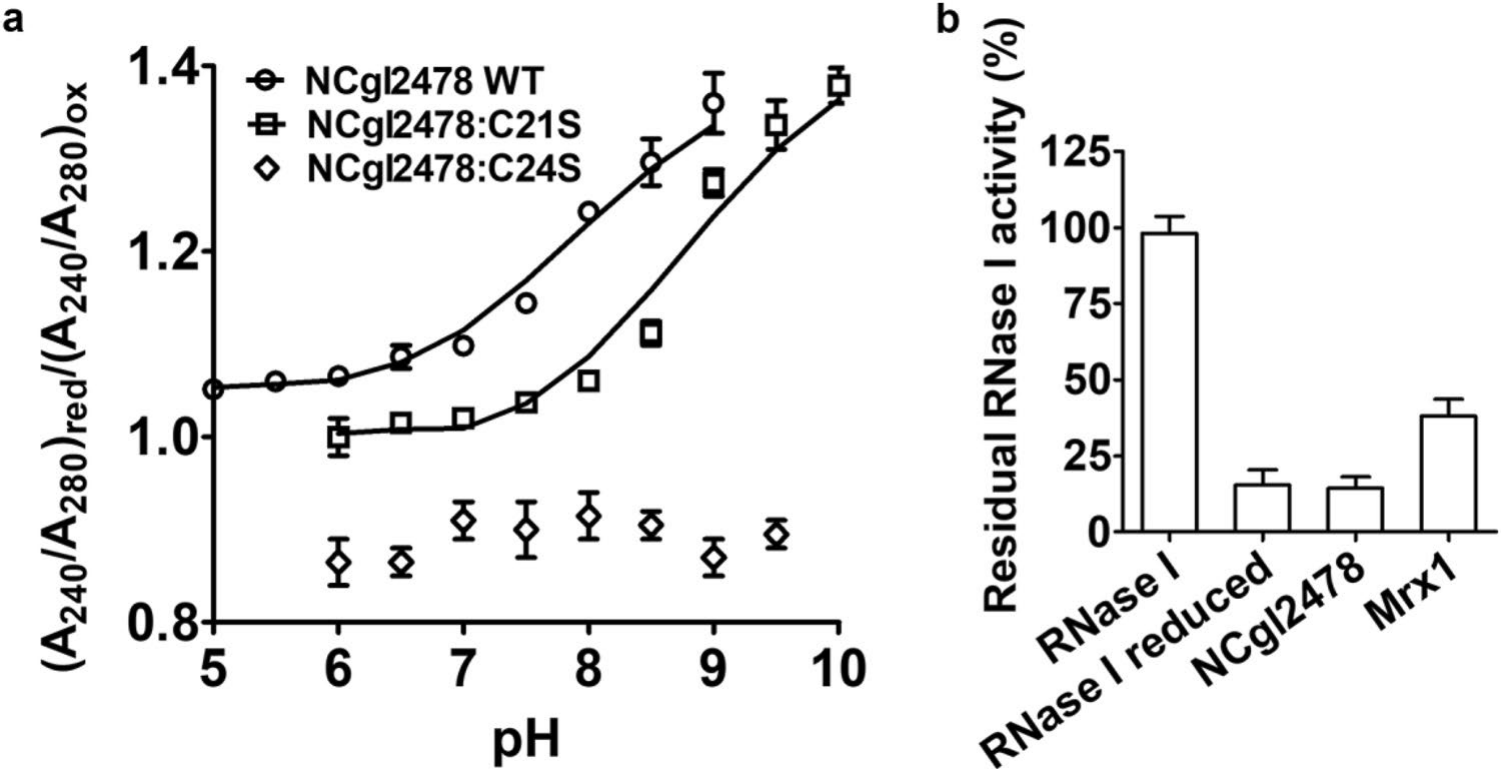
The cysteine of NCgl2478. **a** Cys21 was the nucleophilic cysteine of NCgl2478. The p*K*a measurement of the active site cysteines indicates that Cys21 has an unusual p*K*a value lower than 6 and that Cys24 was the resolving cysteine with a p*K*a of 8.09. Ionized thiol groups (R-SH) extinction coefficient at 240 nm was utilized to measure p*K*a values of cysteines of NCgl2478 WT (○) and the NCgl2478:C24S (□) and NCgl2478:C24S (◊). The ratio composed by 240/280_red_ and 240/280_ox_ in a pH range of 5 to 10 as fitted with the Henderson–Hasselbalch equation. **b** NCgl2478 was not a DsbA-oxidoreductase. The methylene blue RNA intercalating assay was utilized to quantify the activity of RNase I (0.5 μM). *E. coli* RNase I was reduced, and the recovering of activity was monitored in the presence of several enzymes (5 μM NCgl2478 WT or Mrx1). NCgl2478 was unable to catalyze the disulfide bond formation in a previously reduced RNase I. Reactions were performed in duplicate

Since the thiolate ion has a higher absorption at 240 nm wavelength than the thiol group, the p*K*a of active site residues in NCgl2478 was therefore determined by recording the absorption at 240 nm during a pH titration (Roos et al. 2013). As shown in Fig. 4a, the p*K*a values of the nucleophilic Cys21 and the resolving Cys24 were less than 6 and 8.09, respectively. The result indicated that the low p*K*a value made Cys21 function as the nucleophilic Cys. In addition, the p*Ka* value of nucleophilic Cys of NCgl2478 lied between the p*Ka* values of the respective cysteines of Mrx1 (6.8) and DsbA (~ 3.5). Moreover, the p*Ka* of the Cys24 (8.09) was already lower than the p*Ka* of the MSH sulfur (8.76) (Sharma et al. 2016), which made Cys24 more attack Cys21–MSH mixed disulfide, leading to the formation of a Cys21–Cys24 disulfide.

### NCgl2478 has no oxidase properties

To test its putative DsbA-oxidoreductase activity, we used *E. coli* RNase I as a substrate. RNase I was active with its four disulfide bonds correctly formed, making it an ideal model enzyme for oxidative protein folding evaluation (Messens et al. 2007). We used methylene blue intercalated RNA as a substrate to check the RNase activity at 659 nm after the incubation of reduced unfolded RNase I with NCgl2478 and Mrx1 (Greiner-Stoeffele et al. 1996). NCgl2478 did not catalyze the oxidative refolding of RNase (Fig. 4b). Reduced RNase I (unfolded) demonstrated 15.5% of activity relative to folded RNase I (100%). In contrast, in the presence of Mrx1, 38.2% of activity was recovered. Thus, NCgl2478 did not function as an oxidase.

### NCgl2478 reduced mycothiolated mixed disulfides preferably via a monothiol mechanism

*M. tuberculosis* Rv2466c reduced mycothiolated mixed disulfides and intramolecular disulfide bonds coupled to the MSH/Mtr/NADPH pathway via a different mechanism (Rosado et al. 2017). This led us to survey what mechanism NCgl2478 used to reduce mycothiolated mixed disulfides and intramolecular disulfide bonds coupled to the MSH/Mtr/NADPH pathway. The functionalities of NCgl2478 WT, NCgl2478:C21S, and NCgl2478:C24S to reduce insulin and the mixed disulfide between 2-hydroxyethyl disulfide (HED) and MSH (HED–SSM) were tested by following the oxidation of NADPH in the presence of the MSH/Mtr/NADPH system.

**Fig. 5:**
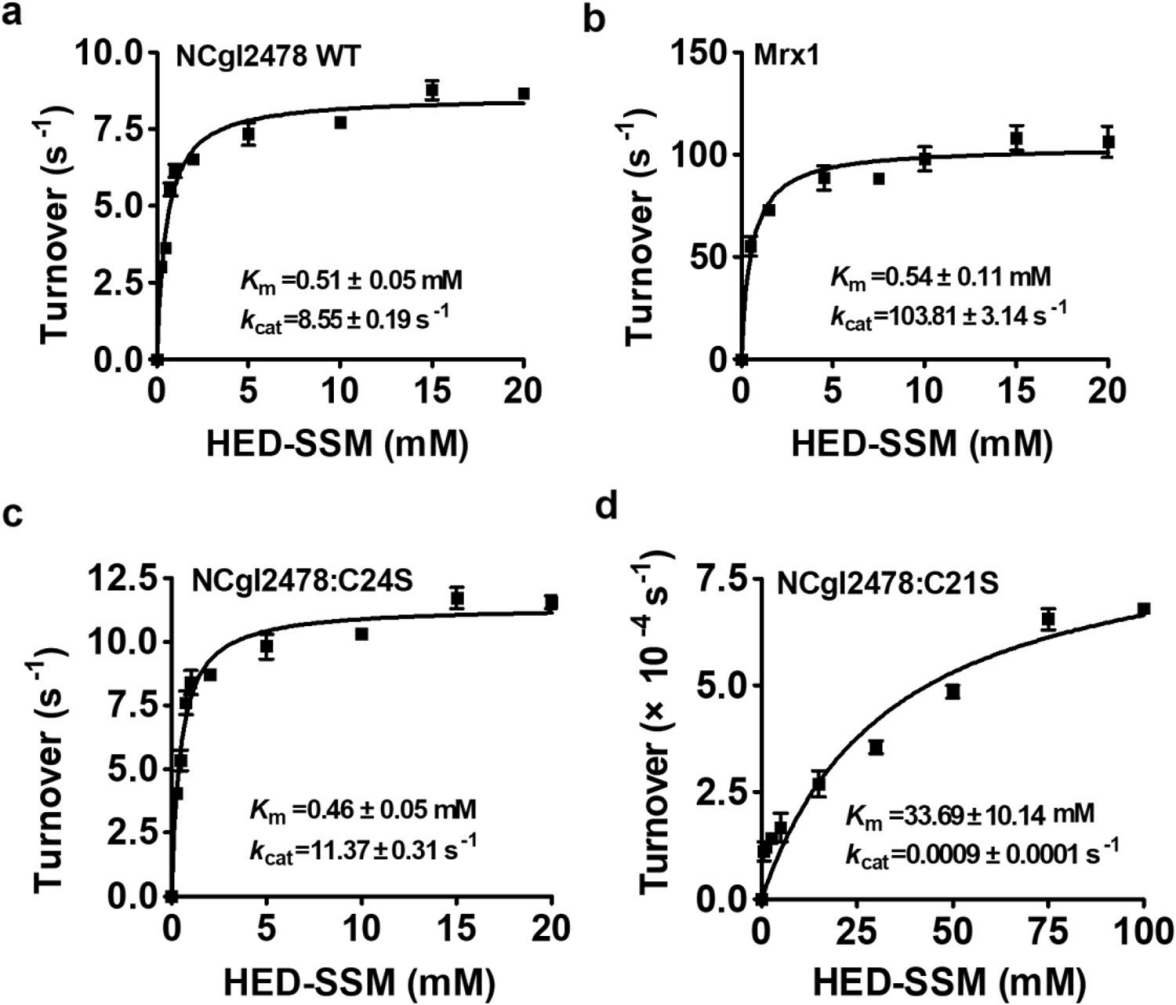
NCgl2478 demycothiolated the mixed disulfide between 2-hydroxyethyl disulfide (HED) and MSH (HED-SSM). Activity of proteins were measured with HED-SSM concentration varying in the range 0–20 mM. The Michaelis–Menten plots of NCgl2478 WT (**a**), Mrx1 (**b**), NCgl2478:C24S (**c**), and NCgl2478:C21S (**d**) activity were calculated using the program GraphPad Prism 5. The data were represented as mean ± SD of three independent experiments

To test the ability of NCgl2478 to reduce mixed disulfides, we compared its reactivity with *C. glutamicum* Mrx1 using HED–SSM as substrates (Fig. 5). We followed NADPH consumption coupled to MSH and Mtr at 340 nm. The *K*_m_ value, *k*_cat_ value, and catalytic coefficient of NCgl2478 or Mrx1 for HED–SSM were calculated to be 0.51 ± 0.05 mM, 8.55 ± 0.19 s^−1^, and 1.68 ± 0.14 × 10^4^ M^−1^ s^−1^, or 0.54 ± 0.11 mM, 103.81 ± 3.14 s^−1^, and 19.22 ± 0.31 × 10^4^ M^−1^ s^−1^, respectively (Fig. 5a, b). NCgl2478:C24S has a slightly higher initial velocity of 11.37 ± 0.31 s^−1^ toward HED-SSM, whereas, the NCgl2478:C21S was inactive. This cysteine substitution experiment indicated that NCgl2478 was functioning under a monothiol mode of action where the nucleophilic Cys21 was required to catalyze mixed disulfide bond reduction. It is worth noting that the activity of Mrx1 was about 11-fold faster in reducing HED-SSM compared with the NCgl2478 enzyme when the MSH/Mtr/NADPH pathway was introduced as an electron donor.

### NCgl2478 reduced intramolecular disulfide bonds via a dithiol mechanism

We further checked the ability of NCgl2478 to reduce intramolecular disulfide bonds in an insulin assay (Table 4). When the MSH/Mtr/NADPH pathway was used as an electron donor, the activity of Mrx1 was 2.1-fold faster in reducing insulin compared with NCgl2478. However, NCgl2478 demonstrated no activity upon mutation of the nucleophilic Cys21 or the resolving Cys24 to serine (Table 4). This led us to conclude that the reduction of insulin occurred via a dithiol mechanism.

**Table 4:** Insulin reduction parameters

Substrates	MSH/Mtr/NADPH				
	Control^a^	NCgl2478	NCgl2478:C21S	NCgl2478:C24S	Mrx1
Rate of precipitation (*A*_600_ 10^–5^ s^−1^) 2.58 ± 0.1	3.14 ± 0.3	12.36 ± 0.05	2.63 ± 0.2	2.93 ± 0.2	25.74 ± 4.1
Starting point (s)	2876	1598	2743	2584	1215

^a^Control, reaction without catalyst

### SigH positively regulated NCgl2478 expression in *C. glutamicum*

**Fig. 6:**
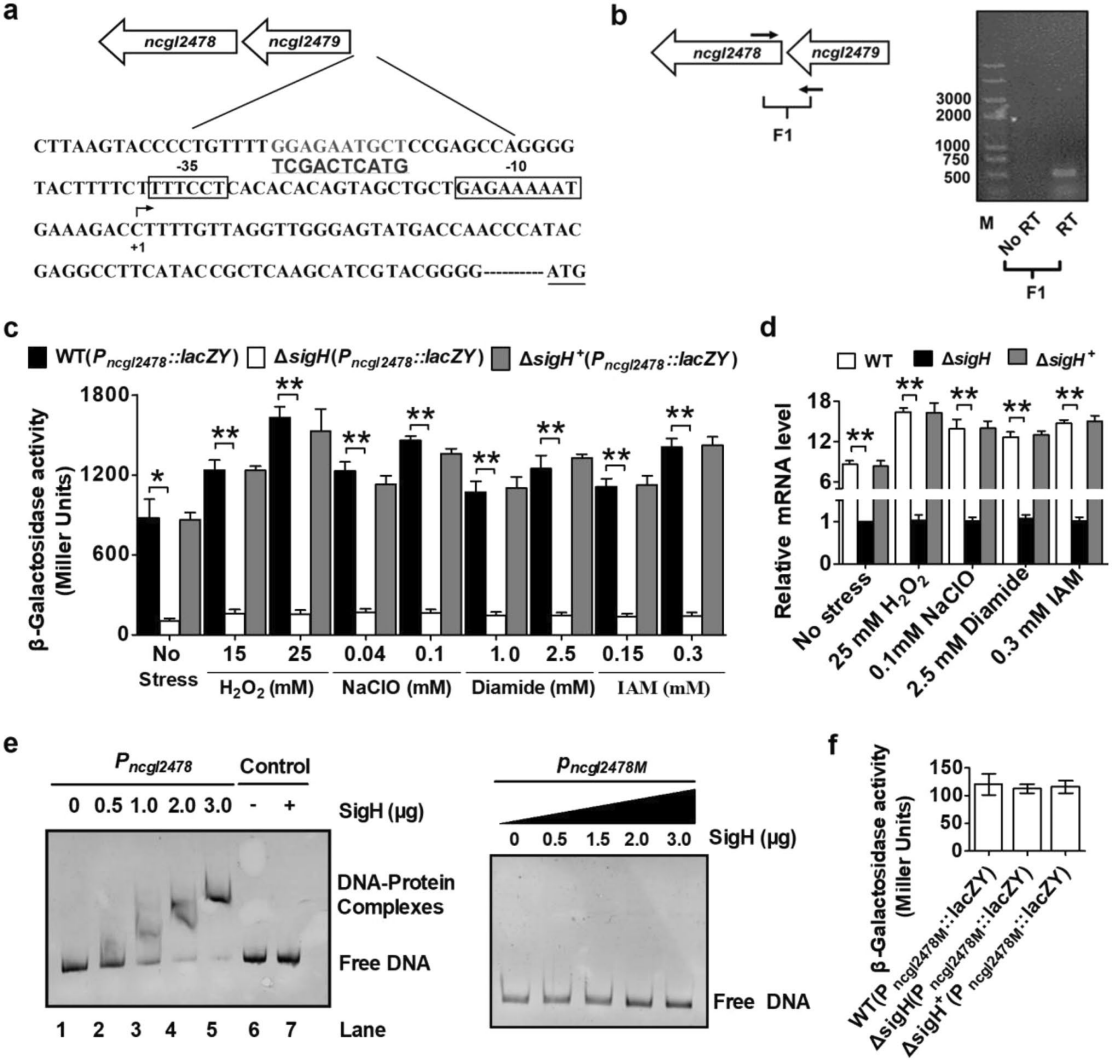
Stress response of *ncgl2478* in *C. glutamicum.* **a** Detailed genetic maps of the regulatory region of NCgl2478. Gray nucleotides indicated the putative binding sites of SigH. The putative transcriptional start site (+ 1) was shown, and the deduced − 35 and − 10 promoter regions were boxed. Underlined sequence with bases in bold showed the mutation of SigH binding site. The start codon was underlined in the *ncgl2478* upstream region. **b** Assays for the *ncgl2478*–*ncgl2479* co-transcription by reverse transcription PCR. The operon structure of *ncgl2478*–*ncgl2479* primer where designed for assays and indicated by blank arrows (left panel). Reverse transcription PCR assays for *ncgl2478*–*ncgl2479* co-transcription (right panel). Negative control PCR reactions omitted the initial reverse transcription step (No-RT). PCR procedure was as follows: reactions were denatured at 95 °C for 50 s, annealed at 58 °C for 40 s, extended at 72 °C for 30 s, and repeated 30 cycles. **c** β-Galactosidase analysis of the *ncgl2478* promoter activity was performed using the transcriptional *P*_*ncgl2478*_*::lacZY* chromosomal fusion reporter expressed in the *C. glutamicum* RES167 parental strain containing empty pXMJ19 (WT). 100 μl of exponentially growing *C. glutamicum* cells treated with different toxic agents at indicated concentrations for 30 min was added to the enzyme reaction system. The values represent the mean results from three independent cultivations, with standard errors. **P ≤ 0.01; *P ≤ 0.05. **d** qRT-PCR assay was performed to analyze the expression of *ncgl2478*. Exponentially growing *C. glutamicum* cells were exposed to different toxic agents at indicated concentrations for 30 min. The levels of *ncgl2478* expression were determined by qRT-PCR. The mRNA levels were presented relative to the value obtained from WT cells without treatment. The values represent the mean results from three independent cultivations, with standard errors. **P ≤ 0.01; *P ≤ 0.05. **e** EMSA was performed to analyze the interactions between His_6_-SigH and the *ncgl2478* promoter (*P*_*ncgl2478*_) or the promoter mutating the identified SigH binding region (*P*_*ncgl2478M*_). A 211-bp fragment amplified from the *ncgl2478* coding region instead of the 211-bp *ncgl2478* promoter (lane 7) and BSA instead of SigH (lane 6) in the binding assays were used as negative controls to determine the binding specificity of SigH. **f** Mutations in the predicted SigH-binding site did not activate the *ncgl2478* expression. Relative levels of transcripts were presented as the mean values ± SD calculated from three sets of independent experiments

Because *C. glutamicum ncgl2478* mutants exhibited sensitivity in the circumstances of various reagents, qRT-PCR, and *lacZY* activity profiling were employed to examine whether *ncgl2478* expression responded to these toxic stress inducers at the transcriptional level. However, no putative promoter was identified upstream of the *ncgl2478* gene. Interestingly, further upstream from *ncgl2478* was *ncgl2479* gene, which is identically orientated as *ncgl2478* (Fig. 6a). Thus, we speculated that *ncgl2478* was organized in a putative operon with *ncgl2479*, which were confirmed to be co-transcribed by reverse transcription PCR (Fig. 6b)*.* Based on the basis of the information, a putative–10 region (GAGAAAAAT) and a putative–35 region (TTTCCT) were identified, which localized within the upstream open reading frame of NCgl2479 (Fig. 6a). According to this phenomenon, we named the promoter DNA fragment of the *ncgl2478-ncgl2479* operon as *P*_*ncgl2478.*_ NCgl2478 expression was investigated by the *lacZY* activity of *P*_*ncgl2478*_::*lacZY* chromosomal promoter fusion reporter and quantitative real-time RT-PCR (qRT-PCR) analysis. As shown in Fig. 6c, the *ncgl2478* expression level was significantly increased in the WT(pXMJ19)(*P*_*ncgl2478*_::*lacZY*) reporter strains treated with various agents, as compared to untreated cells. The result significantly demonstrated that environmental stress induced the expression of *ncgl2478* gene and directly conferred resistance of *C. glutamicum* to stress conditions with the enhancement of expression quantity. A similar pattern of *ncgl2478* expression in response to different reagents was also observed in qRT-PCR analysis (Fig. 6d).

Because Busche et al. found NCgl2478 was one of the main targets of the stress-responsive extra-cytoplasmic function-sigma (ECF-σ) factor SigH by microarray analysis, which is strongly linked to the oxidative stress response in *C. glutamicum* (Busche et al. 2012). Therefore, we tested its regulatory capacity for NCgl2478. Based on the basis of the information, a putative SigH-binding site was identified, which localized within the upstream open reading frame of NCgl2479 (Fig. 6a). Moreover, the *ncgl2479* promoter DNA element was highly similar to the known recognition site for SigH (Busche et al. 2012). NCgl2478 regulation was investigated by chromosomal *P*_*ncgl2478*_::*lacZY* fusion reporter and qRT-PCR analysis. As shown in Fig. 6c, Δ*sigH* (strains lacking *sigH* gene contained empty pXMJ19) significantly decreased the *lacZY* activity of the *ncgl2478* promoter, almost fully recovered by introducing a plasmid pXMJ19 expressing wild-type *sigH* in deletion of *sigH* gene in *C. glutamicum* (∆*sigH*^+^, Δ*sigH* was complemented with plasmid pXMJ19 carrying the wild-type *sigH* gene). These results demonstrated that *C. glutamicum* NCgl2478 was positively regulated by SigH. The positive regulation of *ncgl2478* by SigH was also confirmed by qRT-PCR, with the observation that the mRNA levels of *ncgl2478* were reduced in the Δ*sigH* mutant and restored to the wild-type level in the complemented strain ∆*sigH*^+^ (Fig. 6d). To further determine whether SigH regulated *ncgl2478* expression directly, we examined the interaction between SigH and the *ncgl2478* promoter using an electrophoretic mobility shift assay (EMSA). Incubation of a 211-bp DNA element containing the *ncgl2478* promoter (*P*_*ncgl2478*_) sequence [− 993 to − 782 relative to the ATG start codon of the *ncgl2478* open reading frame (ORF)] with His_6_-SigH led to the formation of DNA–protein complexes, and the abundance of such complexes depended on the amount of SigH (Fig. 6e left panel). However, both BSA instead of His_6_-SigH and a 211-bp control DNA fragment amplified from the *ncgl2478* ORF region showed no detectable binding (Fig. 6e, lanes 6 and 7). To further verify the predicted SigH binding site, the 211-bp promoter DNA containing the mutagenesis sequence of the predicted SigH-binding site (*P*_*ncgl2478M*_), the start and stop sites of which were the same as those of a 211-bp DNA element containing the *ncgl2478* promoter (*P*_*ncgl2478M*_) sequence was synthesized by Shanghai Biotechnology Co., Ltd. and used (Fig. 6a). As shown in Fig. 6e right panel, mutations in the predicted SigH-binding site disrupted the formation of such complexes. Moreover, promoter DNA mutations in the predicted SigH-binding site caused the extremely low transcription activity of *ncgl2478* in WT and Δ*sigH*^+^ strains, similar to that in the Δ*sigH* mutant (Fig. 6f). Thus, SigH directly activated the expression of *ncgl2478* by specifically recognizing an operator within the *ncgl2478* promoter region.

## Conclusion

In this study, we revealed a novel MSH-dependent oxidoreductase NCgl2478 by physiological and biochemical analysis. NCgl2478 promoted *C. glutamicum* resistance to oxidative stress. Its physiological roles in resistance to oxidative stresses were corroborated by its induced expression under various stresses, regulated directly by SigH. Despite its high sequence similarity to DsbA, NCgl2478 did not act as an oxidase. NCgl2478 was less specific in receiving electrons, because both the MSH/Mtr/NADPH and the Trx/TrxR/NADPH pathways regenerated oxidized NCgl2478. NCgl2478 preferentially linked to the MSH/Mtr/NADPH electron pathway via monothiol mechanism to reduce *S*-mycothiolated mixed disulfides. NCgl2478 had a lower initial velocity toward HED-SSM. Thus, NCgl2478 had a similar mode of action, including enzymatic rate, substrate preference, and cell survival under stress, as the previously characterized DsbA–Mrx1 proteins (*M. tuberculosis* Rv2466c and *C. glutamicum* NCgl2339)*.* Together, our work has uncovered NCgl2478 as a member of the novel DsbA–Mrx1 cluster promoted *C. glutamicum* resistance to oxidative stress.

## Data Availability

The study investigators have full access to the article datasets.
